# Phenolic, Antioxidant, Antimicrobial, and *In-vivo *Wound Healing Properties of *Potentilla erecta* L. Root Extract in Diabetic Rats

**DOI:** 10.22037/ijpr.2019.112411.13742

**Published:** 2020

**Authors:** Kaan Kaltalioglu, Barbaros Balabanli, Sule Coskun-Cevher

**Affiliations:** a *Department of Occupational Health and Safety, Vocational School of Espiye, Giresun University, 28600, Giresun, Turkey. *; b *Department of Biology, Faculty of Science, Gazi University, 06500, Ankara, Turkey.*

**Keywords:** Antimicrobial, Diabetes, Phenolics, Potentilla erecta, Wound healing

## Abstract

As stated in many ethnobotany studies, *Potentilla *genus is traditionally used in the treatment of wound healing. In this study, we aimed to investigate to time-course effects of the methanolic extract of *Potentilla erecta (P. erecta) *(MEPE) on diabetic wounds. The subject of the experiments was 36 Wistar rats, divided into three main groups: non-diabetic control (NDM), diabetic control (STZ-DM), and *P. erecta*-treated (MEPE). Diabetes was induced by streptozotocin (STZ). Full-thickness excisional skin wounds were opened in rats. The wounds were treated with *P. erecta* root extract in the MEPE groups. The wound area, wound contraction rate, collagen, thiobarbituric-acid reactive substances (TBARs), nitric oxide (NOx), and glutathione (GSH) levels in wound tissue were determined for the evaluation of the wound healing on days 0, 3 and 7. Phenolic compounds of MEPE were determined by RP-HPLC-UV. The antioxidant properties were spectrophotometrically determined and the antibacterial properties were tested using the microwell-dilution method. Our results demonstrated that MEPE significantly increased wound contraction rate compared to the STZ-DM group on days 3 and 7. MEPE treated rats showed a statistical increase in the levels of NOx, GSH, collagen and a statistical decrease in the levels of TBARs. Our results, for the first time, may indicate that *P. erecta* root extract improves and accelerates diabetic wound healing and also alters oxidative events.

## Introduction

*Diabetes mellitus* (DM) is a metabolic disease characterized by hyperglycemia resulting from abnormal insulin secretion and/or action (1). Impaired wound healing and chronic wounds are important complications of diabetes and cause severe problems, including social and economic. The underlying mechanism of the impaired healing process is not fully understood, but it is thought to be caused by neuropathy, peripheral vascular diseases, abnormal cellular activities, infection, and altered oxidative balance (2–4). Considering that DM affects hundreds of millions of people around the world, it can be said that treatment of impaired or non-healing wounds is very crucial. 

*Potentilla* is a genus of the family *Rosaceae* that consists of about over 500 species of annual and perennial plants (5). *Potentilla* genus, the cinquefoils, are used in the traditional treatment of wounds, burns, bleeding, and stomach and digestive disorders in various countries (6–11). These plants are heavy in secondary metabolites and have anticonvulsant anticancer and antioxidant properties owing to their chemical structures (5). *P. erecta* L. is commonly used as herbal medicine and is called “Kurt pençesi” in Turkey (12). *P. erecta *has a spectacular rhizome, which is thick, woody, and red inside (13). Tannins, astringent polyphenols, are the major active pharmacological compounds (approx. 20%) of the rhizome, especially agrimoniin (13). Tomczyk and Latté reported that in the ethnobotanical review, the rhizome part of the *P. erecta* was used to treat wounds in various countries (5). Similarly, it has also been mentioned that in the books on herbal remedies, *P. tormentilla* (synonym of *P. erecta*) is traditionally used in wound and ulcer healing (14, 15).

The objective of this study was to compare the diabetic control group with the non-diabetic control group and then examine the time-dependent effects of the *P. erecta* methanol root extract (MEPE) on parameters affected by diabetes. For this purpose, wound area, wound contraction rate, and levels of collagen, TBARs, NOx, GSH on days 0, 3, and 7 were determined in circular excisional wound-induced rats, as well as detecting the phenolic, antioxidant and antimicrobial properties of MEPE.

## Experimental

*Plant Extract Preparation*

Plant samples were collected from Bulancak/Giresun province in Turkey (40°42’18.5 “N, 38°03’15.2 “E) and were identified by Dr. Mustafa Karaköse. A voucher specimen has been deposited at the Herbarium of the Vocational School of Espiye, Giresun University, as ESPH 016. The collected samples root parts were separated and dried at room temperature. The dried, powdered root parts were extracted 100 mL of methanol for 8 h using Soxhlet apparatus. The extracts were then filtered using Whatman Millipore filter paper and the solvents were removed with a rotary evaporator. The obtained extract (MEPE) was divided into two parts. The first part was dissolved in pure water for use in antioxidant and antimicrobial assays. The remaining part was dissolved in methanol for use in reversed-phase high performance liquid chromatographic method (RP-HPLC) with UV detection.

*Phenolic Compounds Determination*

Phenolic compounds were determined by RP-HPLC-UV analysis. This analysis was performed via a binary solvent gradient system using a reversed-phase column (C18 HPLC (150 mm × 4.6 mm × 5 μm), Fortis, UK) with a Thermo Finnigan Surveyor HPLC (Thermo Scientific, USA) (A: 2% acetic acid-distilled water, B: 70% acetonitrile-distilled water, initial condition 5% B and it was increased to 80% B in 50 min). The analysis conditions were set at a column temperature of 30 °C, a mobile phase flow rate of 1.2 mL/min and an injection volume of 10 μL. The eluted 17 standard phenolic compounds (gallic acid, protocatechuic acid, chlorogenic acid,* p*-OH benzoic acid, caffeic acid, vanillic acid, syringic acid, ellagic acid, rutin, *p*-coumaric acid, ferulic acid, myricetin, fisetin, quercetin, apigenin, kaempferol, and isorhamnetin) were detected by comparison at 280 and 315 nm.

*Antioxidant Activity Determination*

The total phenolic content (TPC) of the root extracts was determined according to the Folin-Ciocalteu method using gallic acid standard (16). Firstly, 680 mL purified water, 20 mL stock solution (extract), and 400 mL 0.5 mol/L Folin-Ciocalteu reactive were mixed in a tube. After incubation for 10 min at room temperature, 400 mL Na_2_CO_3_ (10%) was added and this mixture was incubated for 120 min at room temperature. After incubation, samples were measured at 760 nm. The activities were given as mg GAE equivalents/g extract. The ferric reducing antioxidant power assay (FRAP) was assayed according to the method of Benzie and Strain (17). The FRAP assay, which is a procedure for determining the antioxidant capacity, based on the fact that the Fe^3+^ -TPTZ complex is reduced in the presence of antioxidants to form blue complex Fe^2+^ -TPTZ and that this complex gives maximum absorbance at 593 nm. Three mililiter FRAP reactive (TPTZ, FeCl_3_, and acetate buffer) and 100 μL extract or blank sample were added experimental tubes and mixed. After 4 min, samples were determined at 593 nm. The results were compared with the calibration curve (FeSO_4_.7H_2_O) and expressed as μM FeSO_4_.7H_2_O equivalents/g extract. The radical scavenging activity (2,2-Diphenyl-1-picrylhydrazyl (DPPH) assay) was measured spectrophotometrically using DPPH radical (18). Briefly, 50 μL stock solutions (extract) were mixed with 5 mL fresh 0.004% (w/v) DPPH solution. The mixture was incubated for 30 min at room temperature in darkness. After incubation, samples were detected at 517 nm. The radical scavenging activity was given as IC_50_ (mg/mL). All antioxidant activity assays were applied in triplicate.

*Antimicrobial Activity Determination*

 The antimicrobial activities of the root extracts were determined according to a microwell dilution method and the minimal inhibitor concentration (MIC) values were calculated. All tested microorganisms were obtained from the Department of Biology, Faculty of Science at Karadeniz Technical University: *Bacillus cereus (B. cereus)* RSKK 709, *Bacillus subtilis (B. subtilis) *subsp. *spizizenii* ATCC 6633, *Staphylococcus aureus (S. aureus) *ATCC 25923, *Enterococcus faecalis (E. faecalis) *ATCC 29212, *Escherichia coli (E. coli) *ATCC 25922, *Pseudomonas aeruginosa (P. aeruginosa)* ATCC 27853 *Klebsiella pneumoniae (K. pneumonia) *ATCC 700603, *Enterobacter cloacae (E. cloacae)* ATCC 13047, *Yersinia pseudotuberculosis (Y. pseudotuberculosis)* ATCC 911, *Acinetobacter baumannii (A. baumannii)* RSKK 02026, and *Candida albicans (C. albicans)* ATCC 14053. The stock solutions (extract) were prepared with pure water at the last concentration of 40 mg/mL and sterilized by filtration via 0.45 μm Millipore filters. One-hundred microliter stock solution was added to the first well and then serial 2-fold dilutions were made in order to obtain a concentration range from 20 to 0.039 μg/mL in each well-containing nutrient broth. The last two wells were used as a sterility control (containing culture broth plus 100 µL of stock solution, without antimicrobial substance) and a growth control. Each test and growth control wells were inoculated with 5 µL of a bacterial suspension (5 × 10^5^ CFU/well). Experiments were performed in triplicate and the microdilution trays were incubated at 28 or 30 or 37 °C. Bacterial growth was determined by addition of 40 µL of an INT (2-(4-iodophenyl)-3-(4-nitrophenyl)-5-phenyltetrazolium chloride) alcoholic solution (0.2 mg/mL). The trays were again incubated given above for 30 min, and in those wells, where bacterial growth occurred, INT changed from yellow to purple. The MIC was described as the concentration in the well containing lowest compound dose that monitored no growth. Ampicillin, amikacin, and fluconazole were used as standard antibacterial and antifungal agents (19).

*Animals*

This study was approved by the Local Ethics Council of Gazi University for Animal Experiments (G.Ü.ET-15.053). 36 male Wistar albino rats (200-250 g) were used. The animals were maintained under a 12 h light/dark cycle and room temperature and were housed singly and fed with standard rat food and water *ad libitum*. 

*Diabetes Induction and in-vivo Wound Healing Experiment*

The animals were randomly divided into three main groups: non-diabetic control (NDM), diabetic control (STZ-DM), and *P. erecta*-treated (MEPE) (n = 12/group). Diabetes was induced in STZ-DM and MEPE groups by a single dose intraperitoneal administration of streptozotocin (STZ) (60 mg/kg, Sigma-Aldrich, USA) freshly prepared in sodium citrate buffer (0.1 mol/L, pH 4.5). Blood glucose levels were measured 72 h after STZ administration by glucometer (Accu-Chek Performa Nano, Roche Diagnostics, GER), and those with above 250 mg/dL were accepted as diabetic. The animals were deeply anesthetized by intramuscular administration of ketamine HCl (50 mg/kg) and xylazine HCl (5 mg/kg) (Alfamine and Alfazyne, Alfasan, Woerden, Holland). The dorsum of the rat was shaved and sterilized. Six full-thickness excisional skin wounds were opened in all rats by using an 8-mm punch (Acu-Punch; Acuderm, USA). The wounds were topically treated with *P. erecta* root extract (50 mg/kg, dissolved in physiological saline, single daily dose) using a sterile pipette in the MEPE groups. No treatment was applied to the NDM and STZ-DM groups. 

*Wound Area and Wound Contraction Rate Determination*

The wound areas were photographed and measured using the ImageJ software (NIH, USA) during the healing process. The wound contraction rate refers to the wound reduction in the original wound area. This rate was calculated as follows: [(wound area day 0–wound area on day 3 or 7)/wound area day 0] × 100. Values are defined as wound area (mm^2^) and the rate of wound contraction.

*Biochemical Estimations*

On days 3 and 7, six animals from each group were killed with intracardiac blood aspiration under anesthesia to collect wound tissue, stored at − 80 °C. At the same time, skin punches indicating 0 days were also collected from non-wounded animals of NDM and STZ-DM groups. Lipid peroxidation was determined spectrophotometrically according to the method detailed before at 535 nm (20). The reactive nitrogen oxide species (NOx) levels were determined spectrophotometrically at 540 nm by Griess reaction (21). Levels of glutathione (GSH) were measured spectrophotometrically according to a modified Ellman method at 412 nm (22). The total amount of collagen (type 1-5) in the wound tissues was determined using a commercial kit (Sircol Collagen Assay Kit, Biocolor, UK) following the manufacturer’s instructions and in the manner specified by Tsuda *et al.* (23). The amount of collagen in the tissues was calculated as μg/mg tissue.

*Statistical Analysis*

All results were presented as mean ± standard deviation (SD). Statistical data comparison was performed using one-way ANOVA and a *Post-hoc* Tukey test (SPSS v. 16, IBM Inc, USA). *P* < 0.001 was considered statistically significant. 

## Results and Discussion

In diabetic patients, impaired wound healing or non-healing wounds are some of the important factors that decrease the quality of life and increase healthcare costs. Previous studies have reported that diabetic wounds exhibit delayed wound closure, decreased collagen levels, and affected oxidative events (2, 24–26). Medical plants are often used in the treatment of many diseases as well as in wound healing. As mentioned in ethnobotanical studies, the *Potentilla* genus is traditionally used for this purpose, but more scientific data is required to determine its efficacy (5, 14 and 15). The current study showed that *P. erecta* extract has therapeutic effects on the diabetic wound healing process on days 3 and 7 in rats.

**Figure 1:**
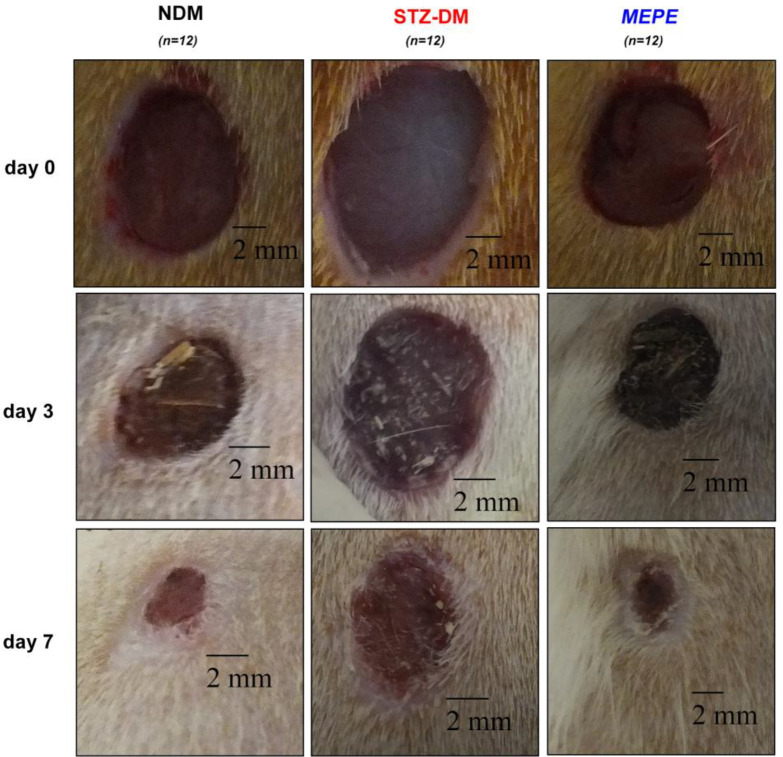
Effects of the methanol extract of *P. erecta* (MEPE) on the wound area

**Figure 2:**
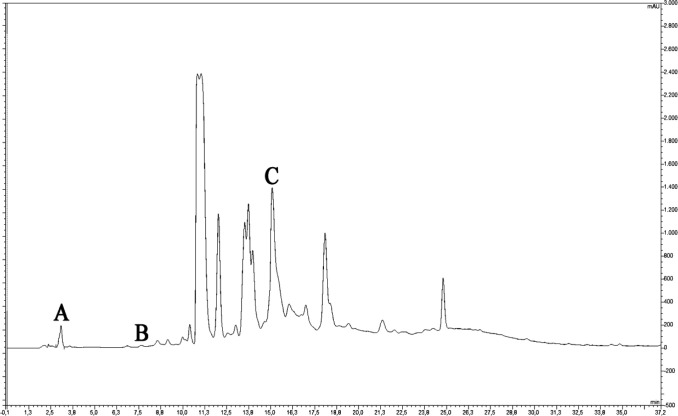
Chromatogram of MEPE (A) gallic acid, (B) protocatechuic acid, (C) rutin

During epithelialization, the epithelial cells at the wound edge migrate towards the wound bed and proliferate to provide wound contraction. Wound contraction is an important indicator of epithelialization and wound starting to heal (27). Delayed or impaired wound healing is a major problem in diabetic patients. In our study, it was determined that the wound area in the diabetic rats was higher than that of the non-diabetic rats (*P* < 0.001), and therefore wound healing was delayed (Table 1 and Figure 1). Topical administration of MEPE, when compared to the diabetic control group (STZ-DM), showed a significant decrease in the wound area on days 3 and 7 (*P* < 0.001). Also, it was observed that the wound area and wound contraction rate in the MEPE group were similar to the non-diabetic control group. Recently, another *Potentilla *species (*P. fulgens*) showed significant wound healing activity, especially on epithelialization (28). Otherwise, gallic acid (0.41 mg/g extract), rutin (0.29 mg/g extract) and protocatechuic acid (0.08 mg/g extract) were identified in the MEPE (Table 2 and Figure 2). The other phenolic compounds of the MEPE determined, but not identified because it is below the limit of detection (LOD). It has been shown that gallic acid and rutin decreased wound area and accelerate wound healing (29, 30). In considering these results, it can be argued that *P. erecta* root extract increases the wound closure of diabetic wound healing to the levels of normal wound healing and contributes to the improvement and acceleration of the diabetic wound healing process.

The collagen is one of the major proteins of the connective tissue and plays a role in cell proliferation and migration as well as providing structural support during the wound healing process (31). In our study, total collagen levels of diabetic rats decreased significantly compared to non-diabetic rats on all days (*P* < 0.001) (Table 3). In many studies supporting our results, it has been reported that collagen and hydroxyproline (a major component of collagen) content decreases in the skin and wound tissues in diabetic patients and diabetes-induced experimental animal models (24, 32). *P. erecta *extract significantly increased total collagen level on the 7^th^ day of healing compared to diabetic control (STZ-DM) group (*P* < 0.001). Similarly, Kundu *et al.* reported that the ethanolic extract of *P. fulgens* increases (another species of *Potentilla*) the hydroxyproline levels in wound tissue (28). Our findings may suggest that the *P. erecta* extract is effective in the proliferation phase of diabetic wound healing process by increasing collagen levels and improves wound healing in diabetic rats.

Phenolic compounds play an active role in the treatment of various diseases by showing antimicrobial and antioxidant activity (33). Infection is one of the important local factors affecting wound healing. The increasing bacteria population in the wound area may delay wound healing (34). MEPE showed antimicrobial activity against *S. aureus, P. aeruginosa, E. coli, *which are pathogens and may cause delayed wound healing (34). However, all determined activities were found to be weak compared to the reference antibiotics (Table 4). Grujić-Vasić *et al.* reported that the various extracts of *P. erecta* showed antimicrobial activity in different degrees against bacteria such as *S. aureus* and *E. coli*, in support of our results (35). These findings suggest that *P. erecta *methanol root extract can help to wound healing by showing antimicrobial activity. Moreover, antioxidant defense systems are very important to protect against reactive oxygen species (ROS)-induced cellular damage. These systems, known as endogenous or exogenous sources, may also contribute positively to the wound healing process (36, 37). Three different methods were used to evaluate the antioxidant properties of MEPE; the amounts of the total polyphenol content (TPC), the ferric reducing antioxidant assay (FRAP) value, and the DPPH scavenging activity are listed in Table 5.

In many studies, cellular damage caused by ROS is determined by lipid, protein, or DNA-induced oxidation products (38). MDA is the end product of lipid peroxidation and the amount at the tissue can be determined by measuring TBARs levels. Compared with the NDM group, TBARs levels were significantly higher in the STZ-DM group on day 0 (*P* < 0.001), but no difference on days 3 and 7. Treatment with MEPE significantly decreased the TBARs levels when compared to NDM and STZ-DM groups on days 3 and 7 (*P* < 0.001) (Table 3). These decreases in TBARs levels may be derived from phenolic compounds of MEPE and its antioxidant activities. FRAP, TPC, DDPH values indicate that MEPE has antioxidant activity (Tables 3 and 5).

Oxygen, reactive oxygen species and reactive nitrogen species are important factors affecting the wound healing process. Sufficient oxygen must be supplied to the wound area for removal of bacteria, angiogenesis, and collagen synthesis (39). In addition, ROS and RNS may serve as secondary messengers and contribute to the healing process (40, 41). Nitric oxide is a highly reactive biological messenger produced via the NOS enzyme by the macrophage, platelet, and fibroblast cells in the wound area (42–44). Nitric oxide may contribute to the improvement of the healing process by enhancing collagen synthesis and storage, angiogenesis, and cell proliferation (45). Witte *et al.* reported that nitric oxide donors could partially reverse the delayed wound healing caused by diabetes (46). We found that NOx levels in diabetic wound tissues reduced compared to non-diabetic wound tissues (*P* < 0.001), but then this decreased level elevated to non-diabetic healing level with MEPE administration on days 3 and 7 (Table 3). Ugusman *et al.* found that rutin (determined in MEPE) promotes NO production via eNOS in cultured human umbilical vein endothelial cells (HUVEC) (47). A strong relationship between NOx, collagen, and fibroblasts has been reported in the literature. Witte *et al.* reported that NO enhances collagen synthesis in fibroblast cell culture (48). Furthermore, fibroblasts first show up in significant amounts in the wound on day 3 after injury and achieve peak amounts around day 7 (49). When the increase of the collagen and the NOx levels on day 7 were evaluated together, *P. erecta *methanol root extract may have triggered synthesis or deposition of collagen by increasing NOx level. 

Glutathione, a low molecular weight endogenous antioxidant, is a highly effective tripeptide in the protection of oxidative stresses in cells and tissues (41). Compared with the NDM group, GSH levels were significantly lower in the STZ-DM group on all days (*P* < 0.001) (Table 3). Rasik and Shukla and Dinçer and Gülen reported that GSH levels in diabetic wound tissues decreased compared with healthy (non-diabetic) wound tissues (50, 51). A statistically significant increase in GSH levels was determined in the MEPE group when compared with the STZ-DM group on days 3 and 7 (*P* < 0.001) (Table 3). Kundu *et al.* showed that *P. fulgens* significantly increased GSH levels in the wound tissues (28). Rutin, chlorogenic acid, caffeic acid, and ferulic acid (determined in MEPE) increases cellular GSH content under *in-vivo *and *in-vitro* conditions (52–55). Based on these studies, it can be considered that the *P. erecta* extract increases the antioxidant capacity of the wound tissue by GSH and consequently positively contributed to the wound healing process.

**Table 1:** Wound areas (mm^2^) and the rates of wound contraction of rats

**Post wounding (days)**	**NDM (n = 12)**	**STZ-DM (n = 12)**	**MEPE (n = 12)**
0	62.09 ± 3.50	62.89 ± 3.73	60.30 ± 1.44
3	29.84 ± 3.17^a^ (52.00 ± 3.28%)	43.80 ± 2.05^a, b, e^ (30.07 ± 6.30%)	30.77 ± 2.11^a, b, d^ (48.92 ± 4.17%)
7	7.04 ± 1.15^a, c^ (88.57 ± 2.38%)	24.07 ± 1.68^a, b, c, e^ (61.55 ± 4.20%)	8.02 ± 0.99^a, b, c, d^ (86.67 ± 1.94%)

Data are expressed as means ± standard deviations.

^ a^ *P* < 0.001 as compared with the non-diabetic control (NDM) on day 0.

^b^*P* < 0.001 as compared with the diabetic control (STZ-DM) group on day 0.

^c^*P* < 0.001 when compared intragroup day 3.

^d^*P* < 0.001 as compared with the diabetic control (STZ-DM) group on same day.

^e^*P* < 0.001 as compared with the non-diabetic control (NDM) group on same day.

**Table 2:** Phenolic constituents of the MEPE by RP-HPLC-UV

	**Phenolic compounds**	**mg phenolic/g extract**
1	Gallic Acid	0.411
2	Protocatechuic Acid	0.082
3	*p*-OH Benzoic Acid	< LOD
4	Chlorogenic Acid	< LOD
5	Vanillic Acid	< LOD
6	Caffeic Acid	< LOD
7	Syringic Acid	< LOD
8	Ellagic Acid	< LOD
9	*p*-coumaric Acid	< LOD
10	Rutin	0.287
11	Ferulic Acid	< LOD
12	Myricetin	< LOD
13	Fisetin	< LOD
14	Quercetin	< LOD
15	Apigenin	< LOD
16	Kaempferol	< LOD
17	Isorhamnetin	< LOD

< LOD: below the limit of detection.

**Table 3:** Effects of the MEPE on some important biochemical parameters during wound healing process on days 0, 3, and 7

		**Collagen** **(µg/mg tissue)**	**NOx** **(nmol/g tissue)**	**TBARs** **(µmol/g tissue)**	**GSH** **(µmol/g tissue)**
NDM (n = 12)	0 day (non-diabetic normal skin)	34.73 ± 1.37	776.64 ± 76.71	57.12 ± 5.02	5.53 ± 0.41
	3 day (n = 6)	18.04 ± 1.32^a^	773.76 ± 58.59	43.19 ± 3.51^a^	2.78 ± 0.15^a^
	7 day (n = 6)	27.21 ± 0.53^a,c^	586.56 ± 47.70^a,c^	47.18 ± 2.61^a^	1.47 ± 0.05^a,c^
STZ-DM (n = 12)	0 day (diabetic normal skin)	15.93 ± 0.90^a^	526.08 ± 31.27^a^	68.14 ± 5.25^a^	0.98 ± 0.98^a^
	3 day (n = 6)	7.26 ± 0.53^a,b,e^	522.24 ± 36.55^a,e^	41.92 ± 3.13^a,b^	0.81 ± 0.07^a,e^
	7 day (n = 6)	7.26 ± 0.60^a,b,e^	555.84 ± 10.14^a^	44.01 ± 3.86^a,b^	0.93 ± 0.03^a,e^
MEPE (n = 12)	3 day (n = 6)	7.25 ± 0.51^a,b,e^	837.12 ± 44.72^b,d^	15.99 ± 1.70^a,b,d,e^	1.27 ± 0.08^a,d,e^
	7 day (n = 6)	26.86 ± 1.93^a,b,c,d^	716.16 ± 35.44^b,d^	16.44 ± 1.71^a,b,d,e^	2.46 ± 0.16^a,e,b,c,d^

Data are expressed as means ± standard deviations.

^a^*P* < 0.001 as compared with the non-diabetic control (NDM) on day 0.

^b^*P* < 0.001 as compared with the diabetic control (STZ-DM) group on day 0.

^c^*P* < 0.001 when compared intragroup day 3.

^d^*P* < 0.001 as compared with the diabetic control (STZ-DM) group on the same day.

^e^*P* < 0.001 as compared with the non-diabetic control (NDM) group on same day.

**Table 4.** Minimal inhibitor concentrations (MIC, μg/mL extract) of plant extract and reference antibiotics

	**MEPE**	**Ampicillin**		**Amikacin**	**Fluconazole**	
* Gram ^+^ ** bacteria**						
*B. cereus *	625	-	0.49			-
*B. subtilis subsp. spizizenii *	2500	0.98	0.49			-
*S. aureus *	625	0.49	0.98			-
*E. faecalis *	1250	1.95	62.5			-
* Gram ^-^ ** bacteria**						
*E. coli *	1250	7.81	0.49			-
*P. aeruginosa *	5000	-	0.49			-
*K. pneumonia *	1250	-	0.49			-
*E. cloacae *	2500	-	0.98			-
*Y. pseudotuberculosis *	156.25	125	31.25			-
*A. baumannii *	5000	7.81	0.98			-
* Fungi						
*C. albicans *	5000	-	-			1.95

**Table 5:** Total phenolic contents, ferric reducing antioxidant power, and radical scavenging activities of MEPE

	**MEPE**
TPC (mg GAE/g extract)	339.467 ± 12.563
FRAP (mM FeSO_4_.7H_2_O equivalents/g extract)	5.080 ± 0.005
DPPH (IC_50_: μg/mL)	0.012 ± 0.001

## Conclusion

Our results demonstrated that contraction in the wound tissues of *P. erecta* treated rats significantly increased compared to diabetic control. *P. erecta* treated rats showed an increase in the levels of NOx, GSH, and collagen and a decrease in the levels of TBARs. Considering biological activities, morphological and biochemical analyzes, for the first time, may indicate that *P. erecta* methanol root extract improves and accelerate healing and alter oxidative events in the diabetic wound healing process in support of ethnobotanical studies. 
